# Kinetic Analysis of Digestate Slow Pyrolysis with the Application of the Master-Plots Method and Independent Parallel Reactions Scheme

**DOI:** 10.3390/molecules24091657

**Published:** 2019-04-27

**Authors:** Pietro Bartocci, Roman Tschentscher, Ruth Elisabeth Stensrød, Marco Barbanera, Francesco Fantozzi

**Affiliations:** 1 Department of Engineering, University of Perugia, Via G. Duranti 67, 06125 Perugia, Italy; francesco.fantozzi@unipg.it; 2 SINTEF Industry AS, Forskningsveien 1, 0373 Oslo, Norway; Roman.Tschentscher@sintef.no (R.T.); RuthElisabeth.Stensrod@sintef.no (R.E.S.); 3 Department of Economics, Engineering, Society and Business Organization, University of Tuscia, 01100 Viterbo, Italy; m.barbanera@unitus.it

**Keywords:** anaerobic digestion, kinetic model, lignin rich, activation energy, thermogravimetric analysis, pre-exponential factor

## Abstract

The solid fraction obtained by mechanical separation of digestate from anaerobic digestion plants is an attractive feedstock for the pyrolysis process. Especially in the case of digestate obtained from biogas plants fed with energy crops, this can be considered a lignin rich residue. The aim of this study is to investigate the pyrolytic kinetic characteristics of solid digestate. The Starink model-free method has been used for the kinetic analysis of the pyrolysis process. The average Activation Energy value is about 204.1 kJ/mol, with a standard deviation of 25 kJ/mol, which corresponds to the 12% of the average value. The activation energy decreased along with the conversion degree. The variation range of the activation energy is about 99 kJ/mol, this means that the average value cannot be used to statistically represent the whole reaction. The Master-plots method was used for the determination of the kinetic model, obtaining that n-order was the most probable one. On the other hand, the process cannot be modeled with a single-step reaction. For this reason it has been used an independent parallel reactions scheme to model the complete process.

## 1. Introduction

### The Importance of Digestate Slow-Pyrolysis Process

Coupling of anaerobic digestion and pyrolysis in integrated processes has become more and more interesting [1]. Anaerobic digestion is a very promising technology to be adopted for biomasses with important moisture content (at least more than 50%). The residue of the anaerobic digestion process is called digestate and contains ashes and components that cannot be decomposed efficiently by the microbia, which are present in the anaerobic digestor (mainly belonging to the following species: Clostridium, Peptococcus, Bifidobacterium, Desulfovibrio, Corynebacterium, Lactobacillus, Actinomyces, Staphylococcus, Streptococcus, Micrococcus, Bacillus, Pseudomonas, Selemonas, Veillonella, Sarcina, Desulfobacter, Desulfomonas, and Escherichia coli) [2]. Thus, digestate is a lignin rich substrate, which is obtained as a coproduct of anaerobic digestion and can be used as a fertilizer or it can be composted. Digestate can also be used to produce energy through the subsequent steps of solid liquid separation and pyrolysis. The University of Perugia has designed and operated a prototypal pyrolysis plant: The Integrated Pyrolysis Regenerated Plant (IPRP) [3]. To understand how the digestate would react in slow pyrolysis conditions some thermogravimetric tests have been performed at SINTEF Norway laboratories (Oslo site) during the project: “Optimization of catalytic pyrolysis of digestate and sewage sludge” funded by the European Commission through the Brisk2 project. Digestate pyrolysis has already been performed in other plant concepts, like the thermocatalytic pyrolysis plant developed at Fraunhofer Institut, Sulzbach-Rosenberg, Germany [4]. In that case TGA tests were performed with 25 mg of dried digestate in argon atmosphere with a heating rate of 20 °C/min. Major weight loss happens before 400 °C. The peak of weight loss is reported at 320–330 °C. The final charcoal mass at 1000 °C was about 35.3 wt% and no kinetic analysis was performed. In the work of Gomez et al. [5] TGA is used to perform an analysis of the thermal stability of digestate, but also in this case kinetic analysis is not performed. To the authors knowledge there are only two works which perform kinetic analysis of digestate: the work of Otero et al. 2011 [6] and the work of Zhang et al. 2017 [7]. In the work of Otero et al. [6], cattle manure is used as a feedstock in laboratory tests aiming at the characterization of its Biochemical Methane Potential (BMP). The obtained digestate is used for the TGA analysis. In our case the digestate is produced from a real anaerobic digestion plant, which is fed with a mixture of animal manure, energy crops (mainly corn an sorghum), and olive pomace. Thus, it is clear that the pyrolysis behavior is deeply influenced by the nature and composition of the digestate. It has also to noticed that, in the work of Otero et al. [6], two models are used for kinetic analysis: OFW [8,9,10] and Vyazovkin [11]. These two isoconversional models are used to mainly obtain the activation energy (E). No pre-exponential factor is derived. This makes this kind of analysis of biomass kinetics quite limited.

In the work of Zhang et al. [7], corn stover digestate is analyzed to obtain the Activation Energy and then the pyrolysis process is simulated using a distributed Activation Energy Model (DAEM). In this case, the digestate is produced from a starting feedstock (corn) which is quite similar to the one which is also analysed in this study. The approach of this work is different because the final aim is to answer the question: Is it possible to calculate also the pre-exponential factor of the digestate pyrolysis reaction?

In the kinetic study of biomass pyrolysis in fact two problems have to be considered with particular care:

1. First of all, there has been much discussion recently on how to determine correctly the pre-exponential factor in biomass pyrolysis and nowadays there are several approaches that can be used, see also the ASTM norm E698-16 on “Standard Test Method for kinetic parameters for thermally unstable materials using Differential Scanning Calorimetry and the Flynn/Wall/Ozawa Method”, the ASTM norm E1641-16 on “Standard test method for decomposition kinetics by thermogravimetry using the Ozawa/Flynn/Wal Method” and also in References [12,13]. Interesting comments are also reported in Reference [14] on the correct use of the aforementioned norms.

2. Another aspect that should be carefully considered is the thermodynamic calculations, which are often performed using a set of equations based on Eyring’s theory of the activated complex [15]. This approach can be hardly adopted for complex processes like pyrolysis of biomass, which involves many reaction steps, the production of intermediates, and complex mass and heat transfer phenomena.

Dealing with the application of the Master Plots method to the analysis of biomass kinetics one of the most interesting contribution is represented by the work of Sanchez-Jiménez et al. [16], also coauthored by Criado, who was one of the first to apply the Master Plots to the kinetic analysis of non-isothermal data [17]. In Reference [16], the Master Plots method is used with the key goal of identifying clearly the kinetic model (f(α)) of cellulose pyrolysis. Usually isoconversional kinetic models are coupled with the Master Plots method because they basically can be used to find if the requirements for the Master Plots method are met. The main assumption to use the different methods presented in the ICTAC recommendation on kinetic computations [12] to calculate the pre-exponential factor is that of “single-step kinetics”. This assumption can be easily checked with an isoconversional method. In particular, in the study in Reference [16], the random scission kinetic model was found to govern cellulose pyrolysis reaction. In this case the activation energy of cellulose was found to be constant and was estimated to be 191 kJ/mol. On the other hand, in the work of de Carvahlo et al. [18], which deals with the kinetic decomposition of energy cane, applying the Master Plots method, it was found that it was not possible to find a unique kinetic model to describe the experimental data. The most consistent model was found to be F7 (7th order reaction model) for conversion lower than 0.5 and F4 (for 0.5 ≤ α ≤ 0.67), F3 (for 0.67 ≤ α ≤ 0.75) and F2 (for conversion higher than 0.9). Reaction order models are generally based on the fact that the driving force depends on the remained concentration of the reactants. The approach used in de Carvahlo et al. [18] maybe is more appropriate for the specific case of digestate, compared to the approach of Sanchez-Jiménez et al. [16], which is more focused on cellulose pyrolysis. Digestate in fact is a lignin rich subproduct, where lignin concentration prevails on the concentration of cellulose and hemicellulose. Dealing with lignin pyrolysis kinetics, an interesting work is done by Jiang et al. [19]. In this work, a review on previous studies on kinetics of different lignin types is presented (e.g., Kraft lignin, Klason lignin, organosolv lignin, Alcell lignin, etc.). From the results, we infer that there is no agreement at the moment on unique values of activation energy and pre-exponential factor for lignin. Based on the results of other literature works, we can infer two important points:

- If the reaction mechanism is not single-step, we cannot use the Master Plots method to calculate the pre-exponential factor of digestate pyroysis (in that case, a multi-step model based on independent parallel reactions can be used);

- We can use the Master Plots method to have an idea of what is the most probable kinetic model for digestate pyrolysis.

Taking inspiration from References [12,13] and what has been said above, the authors decided to apply the Master-plots method to study the kinetics of digestate and understand how to model it better. This approach has not yet been adopted on digestate pyrolysis.

## 2. Results

### 2.1. TG-DTG Curves

**Figure 1:**
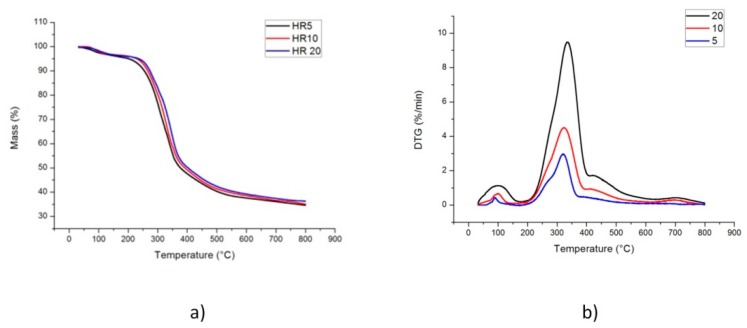
Thermogravimetric (**a**) and Differential thermogravimetric (**b**) curves generated during solid digestate pyrolysis at different heating rates.

Thermochemical decomposition of solid digestate during pyrolysis has been analyzed using thermogravimetric curves, TG, and DTG. Figure 1a shows the weight loss curves obtained during the pyrolysis of solid digestate at different heating rates under inert nitrogen atmosphere. Being a lignocellulosic material, the thermal degradation profile of solid digestate can be divided into three stages, influenced by its chemical and physical composition in terms of hemicellulose, cellulose, and lignin. The first stage started from room temperature and ended at about 180 °C, the mass loss is due to the removal of moisture and the hydrolysis of some extractives [20]. The second stage was the main decomposition region, involving degradation of hemicellulose, cellulose, and a small amount of lignin at a temperature range comprised between 180 °C to 392 °C. The characteristic temperatures of the different stages are shown in Table 1, with the relative standard deviations. It should be considered that to define rigorously Ti, Tf, and Tm the following assumptions have been made:

- Ti represents the temperature at which a conversion of about 5% of the initial mass is obtained;

- Tm represented the temperature at which the maximum conversion is obtained. The average is about 330 °C, which is in agreement with what is reported in Reference [4];

- Tf represents the temperature at which about 80% of the conversion happened.

It is well known that decomposition of hemicellulose, cellulose, and lignin occurs at the temperature range of 160–360 °C, 240–390 °C, and 180–900 °C, respectively [21,22]. Moreover, each DTG curve (Figure 1b) is characterized by a lower temperature shoulder at around 290 °C, corresponding to the decomposition of hemicellulose and a higher temperature peak that can be attributed to cellulose devolatilization. In particular, White et al. [23] pointed out that the cellulose decomposition happens into two ways: (1) Depolymerization with the formation of CO, CO_2_, and carbonaceous residues at low temperature; and (2) integration of bonds at high temperature with the formation of liquid product containing a wide range of organic compounds. After 400 °C, the third stage of pyrolysis began where the slow decomposition of lignin causes the typical long tail of TG-curves. Biochar yield at 800 °C was in the range of 35.08%–36.45%, which was higher than the char yield of other lignocellulosic biomass, such as rice straw (23.68%) and rice bran (25.17%) at 700 °C [24], and camel grass (30.46%) at 550 °C [25], while it was comparable with the char yield of empty fruit bunch (35.14%) at 500 °C [26], reflecting that the lignin content of biomass plays a significant role in biochar formation.

Moreover, as shown in Figure 1a, the shape of the mass loss curve of the solid digestate is not influenced by the heating rate, there is only a little shift to the right in the temperature range from 250 °C to 450 °C, passing from 5 °C/min to 20 °C/min. This result confirms that the degradation chemistry is quite independent from the heating rate and suggests that lower heating rates could be employed in order to optimize the pyrolysis conversion of solid digestate. However, as seen in Figure 1b, DTG curves show an increase in maximum mass loss rates and a slight shift of the major peak to higher temperatures with higher heating rates, mainly due to the combined effects of the heat transfer process at different heating rates and of the kinetics of the thermal volatilization, which result in delayed degradation [27].

### 2.2. Determination of Activation Energy

**Figure 2:**
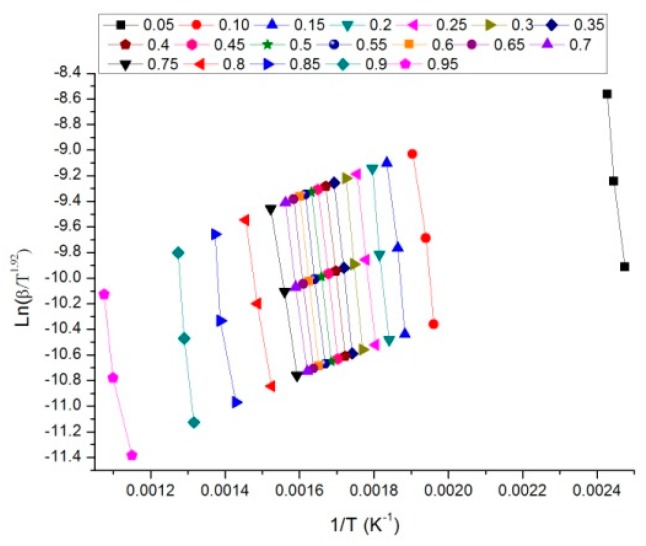
Linear plots in the 0.05–0.95 conversion range for determining activation energy of solid digestate, calculated according to the Starink method.

**Figure 3:**
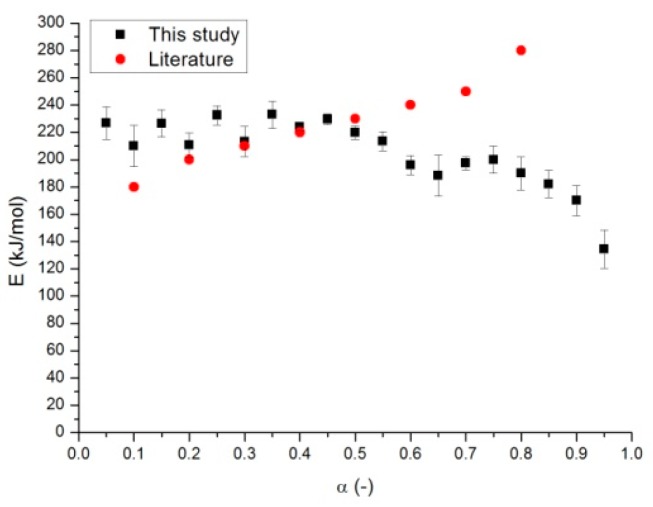
Activation energy distribution (with standard deviations) for solid digestate pyrolysis.

The knowledge of kinetic parameters is essential for effective modeling and design of thermochemical processes because biomass pyrolysis is a heterogeneous reaction that is strongly affected by kinetic parameters, such as: activation energy, pre-exponential factor and kinetic model (also known as kinetic triplet) [16]. Decomposition kinetics during the solid digestate pyrolysis process was calculated using Starink model at degrees of conversion (α) ranging from 0.05 to 0.95 with a step of 0.05 according to the ICTAC recommendations [28]. According to Starink model, the activation energy can be calculated from ln(β/T^1.92^). The plots used for the determination of activation energy at different conversion rates are shown in Figure 2. In particular in the linear plot of ln(β/T^1.92^) versus 1/T the slopes obtained at different conversion rates are equal to -1.0008E/R. Figure 3 presents the values of E and the standard deviation, calculated using the data retrieved from 3 repetitions of the same experiment. The average value of the Activation Energy is about 204.1 kJ/mol, with a standard deviation of 25 kJ/mol, which is about 12% of the average value. The variation range of the Activation Energy is about 99 kJ/mol, which is a high value. This means that the average E value cannot be used to statistically represent the activation energy variation.

**Figure 4:**
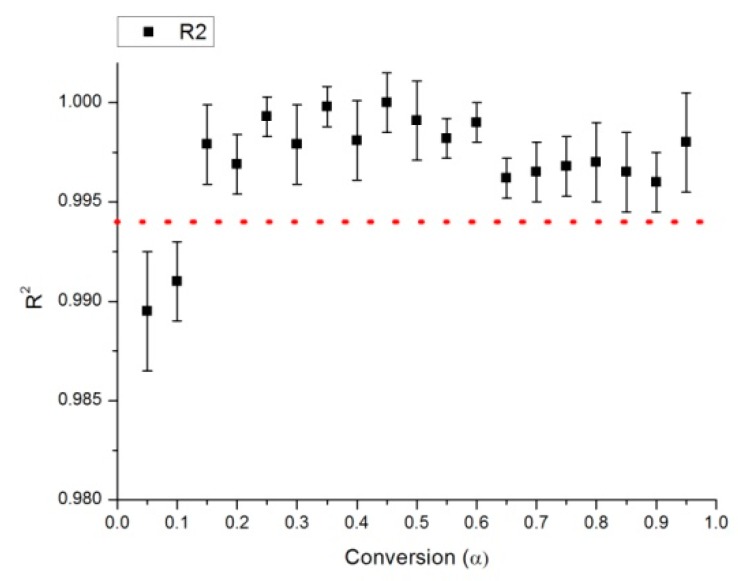
R^2^ coefficient for the calculation of activation energy of solid digestate.

Correlation coefficients (R^2^) are shown in Figure 4. As recommended by the ICTAC Committee [28], since this work was performed with three heating rates, the number of degrees of freedom (calculated as n-2) is only 1, so “in statistical terms, such a plot can be accepted as linear with 95% confidence only when its respective correlation coefficient, R is more than 0.997 (equal to R^2^ of 0.994)”. In our case (see Figure 3), the first two points have a correlation coefficient that is lower than that 0.994. This happened also in the publication of de Carvalho et al. [18] and denotes high uncertainty of the measure activation energies (at least for conversion of 0.05 and 0.1).

The kinetic results show that activation energy is quite dependent on the conversion rate, which means that the pyrolysis of solid digestate is characterized by a complex degradation mechanism that involves different types of reactions, so it cannot be completely considered as a single-step process. The relationship of the activation energy with the conversion rate suggests that the activation energy is almost constant within the conversion range of 0.05–0.55, and then, in the conversion zone of 0.55–0.95, a decline is observed. It can be inferred that there are at least two kinetic models working in sequence. This is mainly due to the fact that digestate is composed by at least four pseudo-components: Cellulose, hemicellulose, lignin, and extractives. Vamvuka et al. [27] reported that the activation energy values for hemicellulose, cellulose, and lignin are in the range of 145–285, 90–125, and 30–39 kJ/mol, respectively. These values are quite accepted in literature at least for cellulose and hemicellulose. For lignin, higher values have also been reported, see Reference [22].

In Figure 3 we see the comparison between the Activation Energy values obtained in this study and the values reported in Literature [7]. The two data sets are not always comparable, especially for higher conversion values. Anyway the decreasing trend of the activation Energy values seems to be more reasonable, also considering other publications on biomass thermal behavior [18] and the composition of the raw material.

### 2.3. Identification of the Reaction Model

The reaction model and the pre-exponential factor are not evaluated directly by the isoconversional methods. Once the activation energy has been calculated the reaction model has to be identified. The identification of a reaction model without a previous verification with the Master-plots model is not recommended, since the solid-state reaction rate can be influenced by diffusion, solid geometry, and reagent concentrations models [18]. Thus in this case, the average value of activation energy (204.1 kJ/mol) is used in the Master-plots method, in order to predict the reaction mechanism of solid digestate. This is an approximation and the method is more accurate when the Activation Energy is constant, but this is done only to have some more hints on the uniqueness of the kinetic model as performed also in [18].

**Figure 5:**
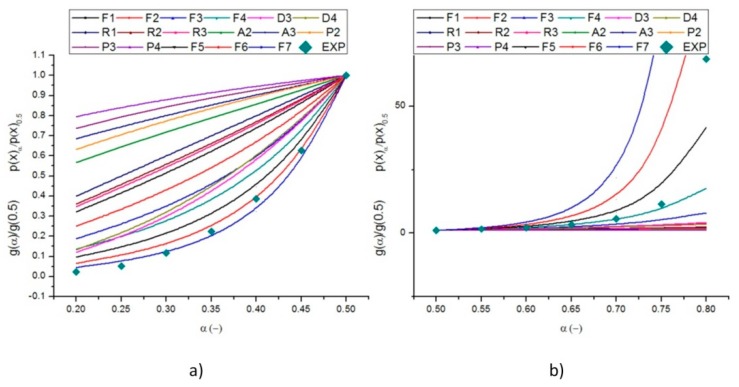
Theoretical and experimental master plots functions from (**a**) 0.2–0.5 and (**b**) 0.5–0.8 of conversion.

Using Equation (9) (see the materials and methods section), the temperature integral, p(x), can be calculated as a function of α by employing the average value of E. Figure 5a,b show the theoretical plots of g(α)/g(0.5) as a function of α, and the experimental plots p(u)/p(u0.5), against α, also the experimental data obtained at β = 10 °C/min, respectively, for α ≤ 0.5 and α ≥ 0.5, are reported. Since the experimental master plots are practically overlapped it was chosen to use in this screening analysis only one heating rate (i.e., 10 °C/min). It can be noted that the Fn model is the most reliable, because the experimental data have the same trends as F4, F5, F6, and F7 models. It is also clear that it is not possible to define any unique function that describes the entire kinetic process for the pyrolysis of solid digestate. The Fn model can be written as:

(1) $$g\left(\alpha \right)=\frac{{\left(1-\alpha \right)}^{1-n}-1}{n-1}$$

It can be noted that the experimental data lie between the theoretical master-plots F6 and F7 for 0.20 ≤ α ≤ 0.50, F4, and F5 for 0.50 ≤ α ≤ 0.75, and F5 and F6 for 0.75 ≤ α ≤ 0.80. It was chosen to refer to the conversion interval 0.2–0.8 because it was thought to be the more stable by the point of view of the pyrolysis reaction. We can conclude that despite the interval takes into account the phases in which the pyrolysis reaction should be more stable, we could not identify a unique theoretical master-plot, which approximates the experimental data perfectly. For this reason, the authors decided to model pyrolysis reaction with an independent parallel reaction scheme. This approach and these types of conclusions are also reported in the work of de Carvahlo et al. [18]. This is the reason why, in the literature, this approach is also gaining more and more interest, see Reference [29].

### 2.4. Independent Parallel Reactions Scheme

**Figure 6:**
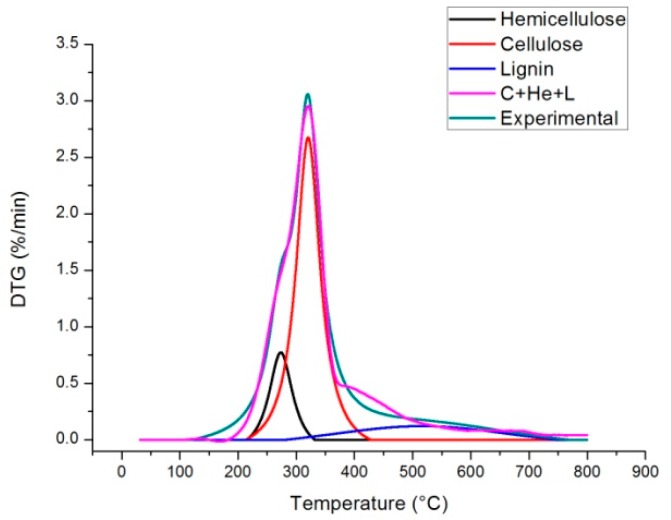
Peak deconvolution result (Heating Rate 5 °C/min).

The results of the peak deconvolution calculations are shown in Figure 6. The heating rate of 5 °C/min is taken as an example, but tests have been performed on all the three heating rates and also repeated three times the final results have been averaged and the standard deviation has been calculated. The fit correlation coefficient (R^2^) between the experimental data and the multi peak fitting result is higher than 0.992 for each of the three considered heating rates.

In Table 2 Activation Energy, Pre-exponential factor and reaction order are presented for each digestate pseudo-component.

Dealing with cellulose Activation Energy, values reported in literature (see [22]) are quite variable and they range from 175 to 235 kJ/mol, our value falls in this range. The pre-exponential factor for cellulose pyrolysis is usually comprised between 1.2 × 10^10^ and 2.2 × 10^19^ min^−1^ [30]. In particular the work of Conesa et al. [30] reports a value of 3.0 × 10^17^ min^−1^, which is quite similar to the one obtained in this study. Dealing with the order of reaction usually a first order reaction is assumed by Antal and Varheghyi [31], and also confirmed in Reference [32].

The values reported in the work of [22] on Activation Energy of hemicellulose pyrolysis are comprised between 149 kJ/mol and 174 kJ/mol. The pre-exponential factor ranges from 10.6 to 15.0 logA/s^−1^. Both values are in agreement with this study. The reaction order is also in agreement with Reference [32].

Dealing with lignin a review of kinetic parameters is reported in the study of Jiang et al. [19]. The reported values for Activation Energy range from 25.2 kJ/mol to 361 kJ/mol, so there is a huge variation. Nevertheless many studies report low values of activation energy and pre-exponential factor for lignin, confirming the results obtained in this study. Thus, if the low values of activation energy and pre-exponential factor can be easily explained, the high standard deviation of the pre-exponential factor indicates that this value in particular has a high level of uncertainty (the same consideration applies to the reaction order).

**Figure 7:**
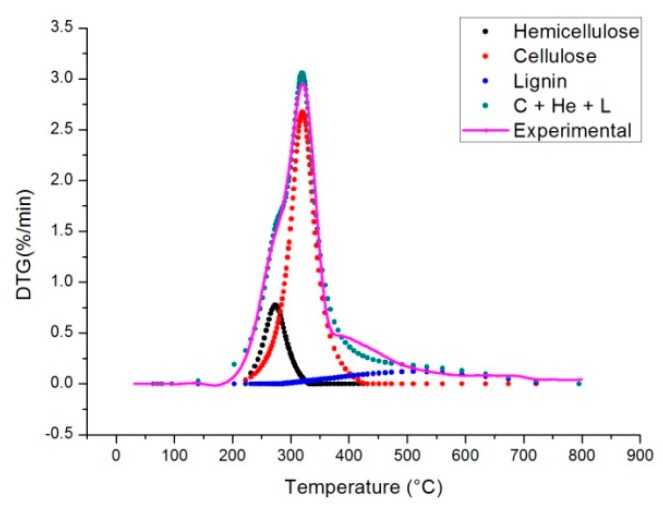
Comparison between experimental DTG data and the combined kinetics of the three-parallel-reaction model (Heating Rate 5 °C/min).

In Figure 7 the comparison between the experimental DTG data and the combined kinetics of the three-parallel-reaction model is shown. This is obtained by integrating the Equation (13) (see Material and Methods section) for each pseudo-component and adding the results to obtain the multi peak trend. To check the quality of the fitting between experimental data and model data Equation (2) is used to calculate the variance (as reported in Reference [28]):

(2) $$S\left(\%\right)=100\;\times \;\sqrt{\frac{\sum_{j=1}^{N}{\left(x_{j}^{c}-x_{j}^{e}\right)}^{2}}{\left(N_{d}-N_{p}\right)}}$$

where j denotes the j-th experimental point; N_d_ denotes the total number of experimental points; Np denotes the total number of unknown parameters (3 in this case); $x_{j}^{e}$ and $x_{j}^{c}$ denote the values of experimental and calculated x, respectively.

The calculated value of S is equal to 0.79%. The correlation coefficient (R^2^) between calculated and experimental data is about 0.990. These values are higher with respect to those shown in the paper of Wang et al. [29]. However, it has to be considered that, compared to the methods used in literature, the one used in this work performs two fitting stages: The first one during peaks deconvolution and the second one when each peak is fitted to a curve finding optimal values of activation energies, pre-exponential factor and reaction order for each pseudo-component. For this reason the method followed in this work is more easy and quick to implement, but probably less accurate. This can also be seen from the correspondence of the blue line shown in Figure 6 with the purple line, there is still space for improving the correlation coefficient and also improve peak deconvolution.

## 3. Materials and Methods

### 3.1. Sample Preparation

The solid digestate used in this study was collected from an on-farm biogas plant of the capacity of 1 MWel located in Central Italy (Umbria Region, province of Perugia), which is fed with a substrate consisting of pig slurry (15 m^3^/d), olive pomace (19 t/d), maize silage (19.6 t/d), sorghum silage (36.4 t/d), and onion scraps (1 t/d). The feedstock is mainly constituted by lignocellulosic biomasses. The set-up of the biogas plant consisted of two anaerobic digesters in parallel (operating at a temperature of 43–44 °C), followed by a post fermenter (operating at a temperature of 37 °C). The solid fraction was obtained by mechanical separation with a screw press separator fed with raw digestate. The sample has been air-dried for 24 h and then oven-dried in a muffle furnace at 105 °C for 8 h. The dried digestate has been ground using an ultracentrifugal mill (mod. ZM200, Retsch) and sieved to obtain a particle size lower than 500 μm. This was done in particular to ensure a heat transfer rate within the kinetic regime of decomposition. The chemical composition of the solid digestate is reported in Table 3.

### 3.2. Experimental Setup

Pyrolysis tests have been carried out to evaluate the rate of mass loss of solid digestate versus temperature, using a thermogravimetric analyzer (NETZSCH STA 449F1, Selma Cloth, Germany). The sample with mass of 20 mg was inserted directly into a small alumina crucible and temperature was ramped from 30 to 800 °C in nitrogen atmosphere with a flow rate of 50 mL min^−1^. According to ICTAC recommendations [28], kinetic experiments should be performed using three to five different heating rates (less than 20 °C min^−1^); therefore, solid digestate was tested at three heating rates of 5, 10, and 20 °C min^−1^. Thermogravimetric (TG) and differential thermogravimetric (DTG) curves were obtained as a function of temperature during each test. Blank tests have been carried out without sample for TG baseline correction in order to avoid any buoyancy effects. All the thermal analyses were repeated for three times to decrease the test error, and the reproducibility was good. The standard deviation on the TG residues is always lower than 0.5 wt%. The experimental thermogravimetric analysis data have been treated, as recommended in Reference [34].

### 3.3. Kinetic Analysis through Iso-Conversional Methods

Biomass pyrolysis is a complex process consisting of several reactions due to the different chemical composition of biomass material. Biomass in general exhibits a three stage pyrolytic reduction with the formation of chars, volatiles and gases [35]. In this study an isoconversional model has been employed for the calculation of the Activation Energy and the Master Plots method is employed for the determination of the reaction model, f(α). The decomposition rate is given by Equation (3) [13]:

(3) $$\frac{d\alpha }{\mathrm{dt}}=k\left(T\right)f\left(\alpha \right)$$

where t is time, T is the absolute temperature, f(α) is the differential form of the reaction model, k(T) is the temperature dependence of the rate constant and α is the conversion degree, expressed as:

(4) $$\alpha =\frac{m_{i}-m_{t}}{m_{i}-m_{f}}$$

where mi is the initial mass of the sample, m_t_ is the mass of the sample at temperature T, m_f_ is the final mass of the sample.

The rate constant k(T) is defined by the Arrhenius equation, Equation (5):

(5) $$k\left(T\right)=\mathrm{Aexp}\left(-\frac{E}{\mathrm{RT}}\right)$$

where E (kJ mol^−1^) is the activation energy, A (s^−1^) is the pre-exponential coefficient and R (J mol^−1^ K^−1^) is the universal gas constant. Then, under non-isothermal conditions at a constant heating rate, β = dT/dt, Equation (3) is transformed into Equation (6):

(6) $$\frac{d\alpha }{\mathrm{dT}}=\left(\frac{A}{\beta }\right)\exp\left(-\frac{E}{\mathrm{RT}}\right)f\left(\alpha \right)$$

The integration of Equation (6) gives Equation (7):

(7) $$\int_{0}^{\alpha }\frac{d\alpha }{f\left(\alpha \right)}=g\left(\alpha \right)=\frac{A}{\beta }\int_{T_{0}}^{T}\exp\left(-\frac{E}{\mathrm{RT}}\right)\mathrm{dT}=\frac{\mathrm{AE}}{\beta R}p\left(x\right)$$

where p(x), with x = E/RT, and g(α) are the temperature integral and the integral form of the reaction model, respectively.

This equation does not present an analytical solution and *p*(*x*) can be obtained by some approximations, depending on the applied kinetic model. In this study, the activation energy was determined employing two kinetic models based on the isoconversional method: Starink [36]. The approximated linear equations of the model are given in Equation (8).

(8) $$\mathrm{Starink}:\;\ln\left(\frac{\beta }{T^{1.92}}\right)=-1.0008\frac{E}{\mathrm{RT}}+\mathrm{constant}$$

The activation energy can be determined by the slope of the regression lines in the graph realized by plotting ln(β/T^1.92^) vs. 1/T for the Starink method.

#### 3.3.1. Master-Plots Method

The Master-plots method was employed for the determination of the reaction mechanism. Moreover, when the value of activation energy is obtained from the isoconversional methods, the Master-plots method allows identifying the reaction model.

The temperature integral, p(x), can be expressed by an approximation. The master-plots method employs Doyle’s approximation [7] to solve the value of p(x):

(9) $$p\left(x\right)=0.00484e^{-1.0516x}$$

In Equation (6), the determination of the pre-exponential factor is affected by the reaction model g(α); therefore, adopting a conversion reference point (α = 0.5) Equation (6) becomes as follows:

(10) $$g\left(0.5\right)=\frac{\mathrm{AE}}{\beta R}p\left(x_{0.5}\right)$$

where x_0.5_ = E/RT_0.5_, T_0.5_ is the temperature at α=0.5 and g(0.5) is the integral form of the reaction model at α = 0.5.

The integral master-plots equation can be obtained by dividing Equation (7) by Equation (10).

(11) $$\frac{g\left(\alpha \right)}{g\left(0.5\right)}=\frac{p\left(x\right)}{p\left(x_{0.5}\right)}$$

In order to determine the reaction model, which better describes the thermal decomposition reaction, the theoretical, g(α)/g(0.5), and experimental, (p(x)/p(x_0.5_), master plots are plotted as a function of the conversion rate. In particular, for a single step decomposition process with a constant g(α) expression, the master-plots method allows to obtain the proper kinetic model with a high degree of certainty [37]. Table 4 shows the most common kinetic functions f(α) and their integral forms g(α).

#### 3.3.2. Independent Parallel Reactions Scheme

To develop an independent parallel reactions scheme for digestate pyrolysis we have based our methodology on the following assumptions:

- The DTG diagram can be decomposed in three peaks representing, respectively: Hemicellulose, cellulose, and lignin. Extractives are considered together with cellulose, because in the differencial thermogram, their presence cannot be easily distinguished;

- To deconvolute the DTG diagram peaks two approaches can be used: Gaussian and Lorentzian. Both approaches can be implemented in Matlab (Mathworks, Natick, MA, USA) and Origin (OriginLab Corporation Northampton, Massachusetts, USA) software. In this case, the second one was used because it gave better results. In fact once that the DTG diagram is decomposed in three curves (see Figure 6), each one is identified by the three parameters that identify the Lorentzian fit curve equation:

(12) $$y=a\frac{1}{1+\left(\frac{x-x_{0}}{\mathrm{dx}}\right)}$$

where a is the amplitude; dx is half width at half maximum (HWHM); x_0_ is the maximum position. 

In this case it was checked that the area of each peak obtained from the deconvolution operation was proportional to the concentration in weight of the pseudo-components (cellulose, hemicellulose, and lignin) inside biomass.

Once the peaks had undergone the deconvolution process, the single peaks (each one corresponding to one pseudo-component) were fitted with the following equation:

(13) $$\frac{{d\alpha }_{\mathrm{theor}}}{\mathrm{dt}}=A*\exp\left[-\frac{E}{\mathrm{RT}}\right]{\left(1-\alpha \right)}^{n}$$

Three variables were calculated for each pseudo-component: Activation Energy, pre-exponential factor and reaction order. The process was repeated for the three heating rates and also for the three repetitions of the experimental tests. The fitting procedure was based on the Matlab patter search tool, which minimized the difference between the deconvoluted values and the calculated values.

(14) $$\mathrm{LSF}=\left\{\overset{3}{\underset{\beta =1}{\sum }}{\left[{\left(d\alpha /\mathrm{dt}\right)}_{\mathrm{deconv}}-{\left(d\alpha /\mathrm{dt}\right)}_{\mathrm{theor}}\right]}^{2}\right\}$$

Concluding, with this method two fitting steps were performed: the first to deconvolute the peaks (based on Lorentz fitting function) and the second to find the optimal activation energy, pre-exponential factor and reaction order for each of the three analysed pseudo-components. The advantage of this method was to avoid fitting the sum of the three Equations (13), corresponding to each pseudo-component, focusing the attention only on one deconvoluted differential curve at a time.

## 4. Conclusions

In this study, the solid fraction of biogas digestate was studied as a potential feedstock for pyrolysis, by analyzing its decomposition kinetics. Pyrolysis of solid digestate comprises three stages. In the first stage the moisture is removed, in the second stage, where the main pyrolysis process happens, where the decomposition of hemicellulose, cellulose, and small amount of lignin occurs. In the third stage, the solid residual is slowly decomposed with the formation of char. Compared to biomass digestate is a lignin rich residue in which the more recalcitrant fractions of cellulose and hemicellulose probably remained, for this reason, a quite high activation energy corresponding to the pyrolysis process has to be noted. The average activation energy determined through the Starink method is about 204.1 kJ/mol, with a standard deviation of 25 kJ/mol, which is about 12% of the average value. The variation range of the Activation Energy is about 99 kJ/mol, which is a high value. This means that the average E value cannot be used to statistically represent the activation energy of the whole reaction. For this reason the application of the Master plots is not fully appropriate in this case and for sure will lead to the obtainment of a value of the pre-exponential factor which is not reliable. So in this case the Master-Plots method was used to have some hints only on the kinetic model. Our study indicated that the most probable thermal degradation mechanism function was the nth order reaction model f(α) = (1 − α)n, with a variable reaction order along with the conversion degree. For this reason, we decided to apply an independent parallel reaction scheme to describe the pyrolysis process of digestate. We chose to apply first a deconvolution process to identify three peaks corresponding to the degradation of the three main pseudo-components in biomass (cellulose, hemicellulos and lignin) and then to apply fitting to the identified peaks. In this way we obtained a less precise estimation of the experimental data, but we sped up the implementation of the model. Some limits of the proposed approach are the following:

- High uncertainty of the activation energies measure (at least in the first conversion values), more tests at different heating rates are required;

- The method has still an important error, especially in the deconvolution phase;

- Master plots method should be used only with single-step reactions;

- In the master plots method all the interval of conversion should be considered (from 0.05 to 0.95);

- In the development of the three independent parallel reactions scheme extractives are considered lumped with cellulose;

- Interactions among the digestate pseudo-components are neglected;

- the pseudo components still have some differences from the real lignin, cellulose and hemicellulose. Especially the kinetic data found for lignin are still uncertain.

## Figures and Tables

**Table 1:** Thermal degradation characteristics of solid digestate at different heating rates.

Heating Rate (°C/min)	Temperature *			DTGmax *
	Ti (°C)	Tf (°C)	Tm (°C)	
5	184 (1)	377 (3)	319 (1)	2.9 (0.5)
10	188 (1)	382 (2)	329 (1)	4.5 (0.7)
20	190 (1)	392 (3)	346 (2)	9.5 (0.9)

* SD values are indicated in brackets.

**Table 2:** Thermal degradation characteristics of solid digestate at different heating rates.

Pseudo-Component	Activation Energy		Pre-Exponential Factor		Reaction Order	
	Value	SD	Value	SD	Value	SD
Cellulose	189 kJ/mol	15 kJ/mol	4.7 × 10^17^ min^−1^	1.5 × 10^16^ min^−1^	1.0	0.1
Hemicellulose	151 kJ/mol	21 kJ/mol	4.4 × 10^14^ min^−1^	5.0 × 10^12^ min^−1^	1.1	0.2
Lignin	64 k/mol	7 kJ/mol	6.3 × 10^3^ min^−1^	1.2 × 10^3^ min^−1^	1.6	1.1

**Table 3:** Characterization of the digestate sample [33].

	Solid Digestate
Proximate analysis (wt.%, dry basis)	
Ash	12.38
Volatile Matter	67.07
Fixed Carbon	20.55
VM/FC	3.29
Ultimate analysis (wt.%, dry basis)	
C	42.52
H	5.94
N	1.79
O	49.75
Compositional analysis (wt.%, dry basis)	
Cellulose	21.64
Hemicellulose	15.08
Lignin	40.88
Extractives	10.02
Calorific value (MJ/kg, dry basis)	
Higher Heating Value	19.74

**Table 4:** Most frequently used mechanism functions and their integral forms [38].

Mechanism	Symbol	f (α)	g (α) *
Order of reaction			
First-order	F_1_	1 − α	−ln(1 − α)
Second-order	F_2_	(1 − α)^2^	(1 − α)^−1^ − 1
Third-order	F_3_	(1 − α)^3^	[(1 − α)^−2^ − 1]/2
Diffusion			
One-way transport	D_1_	0.5α	α^2^
Two-way transport	D_2_	[−ln(1 − α)]^−1^	(1 − α)ln(1 − α) + α
Three-way transport	D_3_	1.5(1 − α)^2/3^[1 − (1 − α)^1/3^]^−1^	[1 − (1 − α)^1/3^]^2^
Ginstling-Brounshtein equation	D_4_	1.5[(1 − α)^–1/3^]^−1^	(1 − 2α/3) − (1 − α)^2/3^
Limiting surface reaction between both phases			
One dimension	R_1_	1	α
Two dimensions	R_2_	2(1 − α)^1/2^	1 − (1 − α)^1/2^
Three dimensions	R_3_	3(1 − α)^2/3^	1 − (1 − α)^1/3^
Random nucleation and nuclei growth			
Two-dimensional	A_2_	2(1 − α)[−ln(1 − α)]^1/2^	[−ln(1 − α)]^1/2^
Three-dimensional	A_3_	3(1 − x)[−ln(1 − x)]^2/3^	[−ln(1 − x)]^1/3^
Exponential nucleation			
Power law, n =1/2	P_2_	2α^1/2^	α^1/2^
Power law, n = 1/3	P_3_	3α^2/3^	α^1/3^
Power law, n = 1/4	P_4_	4α^3/4^	α^1/4^

* g(α) is the integral form of f(α).
